# Appliances Used by Consumers to Prepare Frozen Stuffed Chicken Products — United States, May–July 2022

**DOI:** 10.15585/mmwr.mm7148a2

**Published:** 2022-12-02

**Authors:** Katherine E. Marshall, Michelle Canning, Michael Ablan, Tamara N. Crawford, Misha Robyn

**Affiliations:** ^1^Division of Foodborne, Waterborne, and Environmental Diseases, National Center for Emerging and Zoonotic Infectious Diseases, CDC; ^2^Oak Ridge Institute for Science and Education, Oak Ridge, Tennessee.

Frozen stuffed breaded raw chicken products have repeatedly been implicated in *Salmonella* outbreaks (*1*). These products are partially cooked to set the breading, often making them appear cooked (*2*). Despite their appearance, these products need to be cooked to an internal temperature of 165°F (74°C) to ensure that they are safe to eat. Producers began implementing labeling changes in 2006 to more clearly identify these products as raw; many warn against using microwave ovens (microwaves) to prepare them and provide validated cooking instructions solely for conventional ovens (ovens) (*3*,*4*). However, outbreaks continued to occur after implementation of these labeling changes (*4*). To describe the demographic characteristics of persons who prepare frozen stuffed chicken products and which appliances they use to prepare them, data from a May–July 2022 representative panel survey were analyzed. Although most (82.7%) respondents used an oven as one of their cooking methods, more than one half (54.0%) of respondents also used another appliance, including 29.0% who used a microwave. Oven use was lower among respondents with household income <$25,000 (68.9%), and who lived in mobile homes or other portable types of homes (66.5%). Among respondents who reported using microwaves to cook these products, 8% reported using a microwave with ≤750 W of power, which might be insufficient to thoroughly cook such products (*1*,*5*,*6*). Economic and other factors might influence some groups’ access to recommended cooking appliances. Companies could consider implementing additional interventions that rely less on labeling and consumer preparation practices and focus on controlling or reducing levels of *Salmonella* in these products, such as selling them fully cooked, or monitoring and testing *Salmonella* levels, to ensure safety. These findings highlight challenges consumers might face in preparing frozen stuffed chicken products safely and can guide strategies for regulatory authorities and industry to prevent outbreaks and illnesses associated with them.

During May 31–July 6, 2022, Porter Novelli Public Services conducted the SummerStyles survey using the Ipsos KnowledgePanel. Panel members are recruited nationwide by mail using probability-based sampling by address and are provided with a laptop or tablet and access to the Internet if needed. Among 5,990 members invited to participate, 4,156 (69.3%) completed the survey. Fourteen respondents did not provide responses to the questions of interest, resulting in a final sample of 4,142.

To assess use of cooking appliances to prepare frozen stuffed chicken products, respondents were asked, “What appliances do you use to prepare frozen stuffed chicken products, such as chicken stuffed with broccoli and cheese, chicken cordon bleu, or chicken Kiev?” followed by a list of appliances, or an option to select “I don’t eat these products.” Respondents could select more than one appliance. To assess respondents’ knowledge of their microwaves’ wattage, respondents were asked, “What is the wattage of your household microwave?” To align with the U.S. population distribution, the sample was weighted by sex, age group, household income, race and ethnicity, household size, whether the respondent was the parent of a child or adolescent aged 11–17 years, educational attainment, U.S. Census Bureau region,^†^ and metropolitan status.

Point estimates and 95% CIs were calculated overall and by demographic characteristic (age group, sex, race and ethnicity, U.S. Census Bureau region, household income, highest educational attainment, home type, home ownership, and health insurance status) and compared among respondents who did and did not report preparing frozen stuffed chicken products, and among those who did, the appliances they used and their knowledge of microwave wattage, using Wald chi-square tests. P-values <0.05 were considered statistically significant. All weighted analyses were conducted using SAS (version 9.4; SAS Institute). This activity was reviewed by CDC and was conducted consistent with applicable federal law and CDC policy.^§^

Among 4,142 adults who participated in this survey, 2,546 (61.5%) reported preparing frozen stuffed chicken products (Table 1). A higher percentage of men than women (50.8% versus 44.3%) reported preparing these products as did a higher percentage of younger participants (35.1%) compared with respondents aged ≥60 years (29.1%). A lower percentage of respondents who lived in U.S. Census Bureau West Region (21.8%) reported preparing the products compared with those who lived in other regions (27.4%).

**TABLE 1:** Demographic and socioeconomic characteristics of survey respondents who do and do not prepare frozen stuffed chicken products (N = 4,142) — Porter Novelli Public Services, United States, May–July 2022

Characteristic	Prepares frozen stuffed chicken products, weighted % (95% CI)		
	Yes (n = 2,546)	No (n = 1,596)	p-value*
**Overall**	**61.5 (59.7–63.3)**	**38.5 (36.7–40.3)**	**—**
**Age group, yrs**			
18–29	19.6 (17.3–21.9)	18.0 (15.1–20.9)	0.001
30–44	27.2 (25.1–29.3)	26.0 (23.3–28.8)	0.001
45–59	24.1 (22.2–26.0)	20.9 (18.6–23.2)	0.001
≥60	29.1 (27.2–30.9)	35.1 (32.5–37.6)	0.001
**Sex**			
Female	48.9 (46.6–51.3)	55.3 (52.4–58.2)	0.003
Male	50.8 (48.5–53.2)	44.3 (41.4–47.3)	0.003
Prefer to self-describe	0.2 (0.0–0.4)	0.4 (0.0–0.7)	0.003
**Race and ethnicity**			
AI/AN, NH	0.8 (0.3–1.20)	1.0 (0.3–1.8)	0.082
Asian or NH/OPI, NH	5.7 (4.4–6.9)	7.4 (5.6–9.2)	0.082
Black or African American, NH	11.8 (10.1–13.4)	12.2 (10.0–14.3)	0.082
Hispanic or Latino	18.5 (16.4–20.7)	14.1 (11.7–16.4)	0.082
White, NH	61.7 (59.3–64.1)	64.0 (61.0–67.0)	0.082
Multiple races, NH	1.5 (1.1–2.0)	1.3 (0.8–1.8)	0.082
**Annual household income, US$**			
<25,000	13.5 (11.8–15.3)	11.4 (9.4–13.4)	0.465
25,000–49,999	16.9 (15.1–18.7)	17.2 (14.8–19.5)	0.465
50,000–74,999	16.4 (14.6–18.2)	16.3 (14.1–18.6)	0.465
≥75,000	53.2 (50.9–55.6)	55.1 (52.1–13.4)	0.465
**Highest educational attainment**			
High school diploma or less	38.0 (35.7–40.4)	37.1 (34.1–40.1)	0.374
Some college	27.6 (25.6–29.7)	26.2 (23.6–28.7)	0.374
College graduate or higher	34.3 (32.2–36.5)	36.7 (34.0–39.5)	0.374
**U.S. Census Bureau region^†^**			
Northeast	18.0 (16.2–19.7)	16.1 (14.1–18.2)	0.009
Midwest	21.4 (19.5–23.3)	19.3 (17.0–21.6)	0.009
South	38.8 (36.5–41.1)	37.2 (34.2–40.1)	0.009
West	21.8 (19.8–23.8)	27.4 (24.7–30.1)	0.009
**Housing type**			
One-family house, townhouse, or condominium	79.6 (77.6–81.6)	79.5 (77.0–82.0)	0.933
Building with two or more apartments	16.1 (14.3–17.9)	16.5 (14.2–18.8)	0.933
Other (e.g., mobile home, RV, boat, or van)	4.2 (3.2–5.3)	4.0 (2.8–5.2)	0.933
**Housing ownership**			
Owned	69.6 (67.3–71.9)	71.4 (68.5–74.2)	0.536
Rented	28.4 (26.1–30.6)	27.0 (24.2–29.8)	0.536
Occupied without payment of rent^§^	2.1 (1.4–2.7)	1.6 (0.7–2.5)	0.536
**Health insurance**			
Yes	91.7 (89.7–93.7)	92.1 (89.7–94.5)	0.775
No	8.3 (6.3–10.3)	7.9 (5.5–10.3)	0.775
**Visited primary health care provider during last 12 mos**			
Yes	78.4 (76.4–80.5)	75.2 (72.4–77.9)	0.064
No	21.6 (19.5–23.6)	24.8 (22.1–27.6)	0.064

**Abbreviations:** AI/AN = American Indian or Alaska Native; NH = non-Hispanic; NH/OPI = Native Hawaiian or other Pacific Islander; RV = recreational vehicle.

* The p-value for weighted Wald chi-square test; p-values <0.05 were considered statistically significant.

^†^ https://www2.census.gov/geo/pdfs/maps-data/maps/reference/us_regdiv.pdf

^§^ Housing that is occupied without payment of rent could include housing owned by friends or relatives who live elsewhere and who allow occupancy without charge or could include housing provided as compensation for persons such as caretakers, ministers, tenant farmers, or others.

Overall, 2,107 (82.7%) of the 2,546 respondents reported using an oven as one of the cooking appliances used for preparing frozen stuffed chicken products (Table 2). Oven usage was lower among respondents with an annual household income of <$25,000 (68.9%) than among those with household incomes ≥$25,000 (84.9%; p<0.001), those who completed some college or less (80.4%) than among those who completed college (87.2%; p = 0.0002), respondents living in mobile homes, recreational vehicles, boats, vans, or other types of home (66.5%) compared with those living in a one-family house, townhouse, condominium, or apartment (83.5%; p = 0.014), and among those who occupied their home without payment of rent^¶^ (63.1%) compared with those who owned or rented their home (83.1%; p = 0.037).

**TABLE 2:** Appliances used to prepare frozen stuffed chicken products,* by appliance type and user characteristics (N = 2,546) — Porter Novelli Public Services, United States, May–July 2022

Characteristic	Appliance type										
	Microwave oven		Toaster oven		Air fryer		Appliance not listed		Conventional oven		Total
	No. (weighted %)	95% CI	No. (weighted %)	95% CI	No. (weighted %)	95% CI	No. (weighted %)	95% CI	No. (weighted %)	95% CI	
**Total**	**738 (29.0)**	**26.8–31.1**	**349 (13.7)**	**12.1–15.3**	**755 (29.7)**	**27.5–31.9**	**97 (3.8)**	**2.8–4.8**	**2,107 (82.7)**	**80.8–84.6**	**2,546**
**Age group, yrs**											
18–29	146 (29.3)	23.0–35.5	71 (14.3)	9.5–19.0	186 (37.4)	30.7–44.1	24 (4.9)	1.9–7.9	432 (86.6)	81.7–91.5	**499**
30–44	196 (28.3)	24.1–32.6	98 (14.2)	11.0–17.4	227 (32.8)	28.5–37.1	30 (4.3)	2.4–6.2	563 (81.2)	77.4–85.1	**693**
45–59	160 (26.1)	22.1–30.1	93 (15.2)	11.9–18.5	179 (29.2)	25.1–33.2	28 (4.6)	2.6–6.5	499 (81.1)	77.4–84.9	**614**
≥60	235 (31.7)	28.5–34.9	86 (11.6)	9.5–13.8	162 (21.9)	19.0–24.9	15 (2.0)	1.0–3.0	613 (82.9)	80.1–85.6	**740**
**Sex**											
Female	301 (24.2)	21.2–27.2	142 (11.4)	9.1–13.6	361 (29.0)	25.8–32.2	42 (3.4)	2.1–4.6	1,052 (84.4)	81.8–87.0	**1,246**
Male	437 (33.7)	30.7–36.8	207 (16.0)	13.6–18.4	392 (30.3)	27.3–33.4	55 (4.2)	2.8–5.7	1,051 (81.2)	78.5–83.9	**1,294**
Prefer to self-describe	0 (—)	—	0 (—)	—	2 (36.3)	0.0–78.1	0 (—)	—	4 (74.1)	32.8–100.0	**6**
**Race and ethnicity**											
AI/AN, NH	2 (11.2)	0.0–25.6	3 (13.9)	0.0–38.6	4 (21.5)	0.0–48.7	4 (22.4)	0.0–45.7	15 (77.0)	49.3–100.0	**19**
Asian or NH/OPI, NH	61 (42.4)	31.2–53.7	36 (25.2)	15.6–34.8	62 (43.2)	31.9–54.6	12 (8.6)	2.6–14.6	96 (66.3)	55.5–77.1	**144**
Black or African American, NH	86 (28.6)	21.9–35.3	32 (10.7)	6.1–15.2	101 (33.7)	26.5–40.9	2 (0.7)	0.0–1.5	239 (79.9)	73.6–86.1	**300**
Hispanic or Latino	126 (26.8)	20.9–32.6	63 (13.4)	9.2–17.6	149 (31.6)	25.5–37.7	25 (5.2)	2.0–8.3	371 (78.5)	73.0–84.0	**472**
White, NH	454 (28.9)	26.4–31.4	213 (13.6)	11.6–15.5	430 (27.3)	24.9–29.8	49 (3.1)	2.1–4.1	1,353 (86.1)	84.2–88.0	**1,572**
Multiple races, NH	8 (20.1)	10.5–29.8	2 (4.5)	0.0–9.5	9 (22.4)	12.3–32.6	4 (10.5)	0.0–21.6	33 (84.3)	75.4–93.3	**39**
**Annual household income, US$**											
<25,000	128 (37.2)	30.3–44.0	44 (12.8)	7.9–17.7	115 (33.2)	26.6–39.9	21 (6.0)	2.5–9.5	237 (68.9)	(62.3–75.6)	**344**
25,000–49,999	115 (26.7)	21.5–31.9	59 (13.8)	9.9–17.7	144 (33.4)	27.6–39.2	15 (3.5)	1.1–6.0	350 (81.5)	77.1–86.0	**430**
50,000–74,999	107 (25.8)	20.6–31.0	53 (12.7)	8.7–16.8	99 (23.7)	18.6–28.8	16 (4.0)	1.7–6.2	348 (83.4)	78.5–88.3	**417**
≥75,000	387 (28.6)	25.8–31.4	193 (14.2)	12.0–16.4	399 (29.4)	26.5–32.3	44 (3.3)	2.1–4.4	1,171 (86.4)	84.2–88.6	**1,355**
**Education**											
High school diploma or less	299 (30.8)	26.9–34.7	126 (13.0)	10.2–15.9	298 (30.8)	26.8–34.8	28 (2.8)	1.5–4.2	763 (78.8)	75.3–82.3	**969**
Some college	177 (25.1)	21.3–28.9	92 (13.1)	10.0–16.2	216 (30.7)	26.6–34.8	41 (5.8)	3.3–8.2	582 (82.7)	79.2–86.2	**703**
Completed college or higher	262 (30.0)	26.7–33.4	131 (15.0)	12.4–17.5	241 (27.6)	24.3–30.9	29 (3.3)	2.1–4.5	762 (87.2)	84.7–89.7	**874**
**U.S. Census Bureau region^†^**											
Northeast	113 (24.8)	20.2–29.4	67 (14.6)	10.9–18.3	124 (27.2)	22.1–32.3	13 (2.9)	1.0–4.8	389 (85.0)	81.1–88.8	**458**
Midwest	164 (30.0)	25.5–34.6	65 (11.9)	8.5–15.2	156 (28.6)	24.2–33.1	26 (4.8)	2.7–6.9	455 (83.5)	79.6–87.4	**545**
South	276 (27.9)	24.5–31.4	125 (12.6)	10.0–15.2	297 (30.1)	26.5–33.7	28 (2.9)	1.5–4.2	825 (83.4)	80.5–86.4	**989**
West	184 (33.2)	28.3–38.1	93 (16.7)	12.9–20.6	177 (32.0)	27.0–37.0	29 (5.2)	2.6–7.8	437 (78.9)	74.5–83.3	**555**
**Housing type**											
One-family house, townhouse, or condominium	582 (28.7)	26.4–31.1	266 (13.1)	11.4–14.9	585 (28.9)	26.5–31.3	69 (96.6)	95.6–97.6	1,706 (84.1)	82.2–86.1	**2,027**
Building with two or more apartments	113 (27.4)	22.0–32.8	58 (14.2)	9.9–18.5	132 (32.2)	26.3–38.1	19 (4.5)	1.8–7.2	329 (80.0)	75.1–85.0	**411**
Other (e.g., mobile home, RV, boat, or van)	43 (39.6)	27.0–52.2	25 (22.9)	11.1–34.8	38 (35.0)	22.6–47.4	9 (8.8)	0.4–17.1	72 (66.5)	54.2–78.8	**108**
**Housing ownership**											
Owned	495 (27.9)	25.5–30.4	245 (13.8)	11.9–15.7	506 (28.6)	26.1–31.1	60 (3.4)	2.4–4.4	1,502 (84.8)	82.8–86.8	**1,772**
Rented	216 (29.9)	25.5–34.4	97 (13.4)	10.0–16.7	231 (32.1)	27.5–36.6	35 (4.9)	2.6–7.2	571 (79.1)	75.1–83.2	**722**
Occupied without payment of rent^§^	26 (49.9)	33.1–66.7	8 (15.0)	1.9–28.2	18 (33.3)	17.2–49.4	2 (2.9)	0.0–8.6	33 (63.1)	46.3–79.9	**53**
**Health insurance**											
Yes	432 (28.9)	26.1–31.7	199 (13.3)	11.2–15.5	435 (29.1)	26.3–31.9	61 (4.1)	2.8–5.4	1,255 (83.9)	81.5–86.2	**1,496**
No	41 (30.1)	18.5–41.7	16 (11.8)	4.4–19.2	53 (38.8)	26.3–51.3	4 (3.0)	0.0–6.5	103 (76.2)	65.4–87.0	**136**
**Accessed primary health care provider in last 12 mos**											
Yes	571 (29.3)	26.8–31.7	251 (12.8)	11.1–14.6	576 (29.5)	27.1–32.0	70 (3.6)	2.6–4.6	1,617 (82.9)	80.8–85.0	**1,951**
No	144 (26.8)	21.9–31.7	88 (16.5)	12.4–20.5	163 (30.3)	25.1–35.5	25 (4.7)	2.1–7.2	450 (83.9)	79.6–88.1	**536**

**Abbreviations:** AI/AN = American Indian or Alaska Native; NH = non-Hispanic; NH/OPI = Native Hawaiian or other Pacific Islander; RV = recreational vehicle.

* Such as chicken stuffed with broccoli and cheese, chicken cordon bleu, or chicken Kiev.

**^†^** https://www2.census.gov/geo/pdfs/maps-data/maps/reference/us_regdiv.pdf

§ Housing that is occupied without payment of rent could include housing owned by friends or relatives who live elsewhere and who allow occupancy without charge or could include housing provided as compensation for persons such as caretakers, ministers, tenant farmers, or others.

More than one half (54.0%) of respondents reported preparing frozen stuffed chicken products using appliances other than or in addition to ovens, including air fryers (29.7%), microwaves (29.0%), toaster ovens (13.7%), or another appliance (3.8%). Microwave usage was higher among men (33.7%), respondents with household incomes <$25,000 (37.2%), and those who occupied their home without payment of rent (49.9%), compared with women (24.2%; p≤0.001), respondents with incomes ≥$25,000 (27.7%; p = 0.011), and those who rented or owned their home (28.5%; p = 0.031).

Among 730 respondents who reported using a microwave to prepare frozen stuffed chicken products, approximately one third (34%) did not know the wattage of their microwave (Figure). A higher percentage of respondents aged 18–29 years did not know their microwave’s wattage (46%) compared with respondents aged ≥30 years (31%; p = 0.03). Overall, 8% of respondents who reported preparing frozen stuffed chicken products using a microwave had microwaves with a power level ≤750 W.

**FIGURE:**
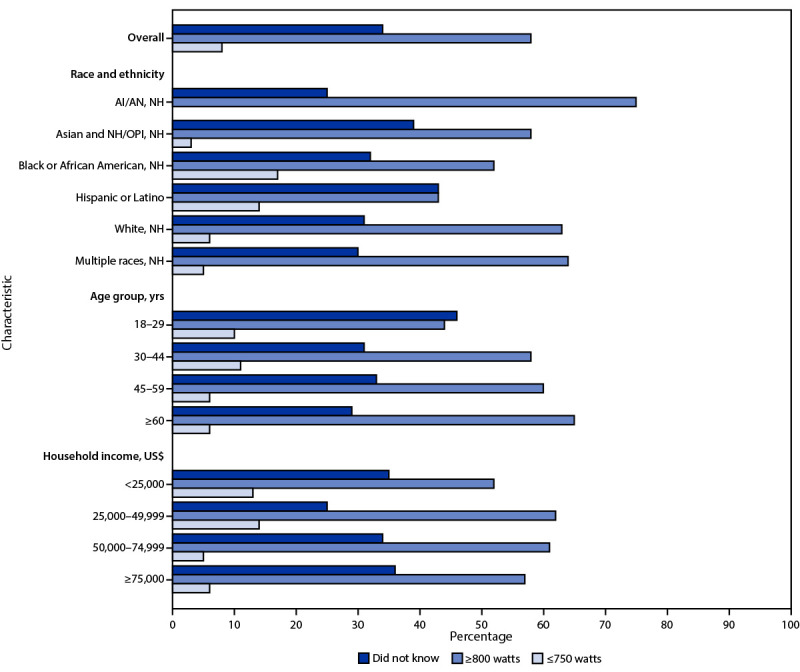
Characteristics of respondents who prepared frozen stuffed chicken products using a microwave oven, by reported microwave wattage (N = 730)* — United States, May–July 2022 **Abbreviations:** AI/AN = American Indian or Alaska Native; NH = non-Hispanic; NH/OPI = Native Hawaiian or other Pacific Islander. * Seven respondents who reported, “I don't have a microwave” when asked about the wattage of their household microwave but reported preparing the product in a microwave were excluded.

## Discussion

Although ovens were the most commonly reported appliance used to cook frozen stuffed chicken products, more than one half of respondents (54.0%) reported using other appliances instead of or in addition to ovens, including microwaves (29.0%), a circumstance that historically has been reported frequently by ill persons in outbreaks associated with frozen stuffed chicken products (*1*). Respondents with lower incomes and who live in mobile types of homes reported lower oven use and higher microwave use. Persons within these groups might be at increased risk for illness related to both challenges in preparing these foods and access to appliances.

Efforts to prevent *Salmonella* infections linked to frozen stuffed chicken products have relied on manufacturers to develop validated cooking instructions and labeling to alert the consumer to which appliances are recommended to cook them (i.e., ovens). Studies indicate that microwaves, air fryers, and toaster ovens inconsistently heat frozen stuffed chicken or frozen raw breaded chicken (*4*,*6*,*7*). Therefore, cooking instructions often do not include information about cooking the product in air fryers or toaster ovens and might warn against using microwaves. However, previous studies have found that some consumers infrequently read package instructions (*8*,*9*), including one report that found some consumers discarded packaging when the products were brought home and never saw cooking instructions (*9*). In this survey, 30% of respondents reported using an air fryer, 29% a microwave, and 14% a toaster oven. These findings suggest that relying on labeling and cooking instructions might not be sufficient to prevent illness. Further, even when cooking these products in an oven, verifying the temperature of the finished product is important (*7*). However, food thermometer usage can be low; one study found that even among persons who owned a food thermometer, only 38% typically used them to check doneness of frozen chicken products (*2*).

Preparing frozen stuffed chicken products in an oven requires access to a working oven. In this survey, persons with lower income, who live in mobile types of homes, and who live in their home without payment of rent reported lower oven use. Persons who live in mobile types of homes might have less or insufficient space for a conventional oven. Appliances like microwaves are small, often portable, and cost less to own and operate than an oven. These findings suggest that economic and other factors might influence some groups’ access to recommended cooking appliances.

Barriers to using ovens, combined with the convenience of microwaves’ shorter cooking times, might encourage consumers to use microwaves. Microwaves require adjusting cooking times based on the microwave’s wattage. Consumers who do not know their microwave’s wattage, as was the case among approximately one third of respondents in this survey, might not be able to adjust cooking times and might therefore be less likely to prepare these products safely. In addition, 8% of all respondents who reported using a microwave to prepare these products and knew the wattage had microwaves with a power level ≤750 W. Studies suggest that lower wattage microwaves might be insufficient to cook these products (*1*,*5*,*6*).

Current measures to prevent *Salmonella* infections linked to contaminated frozen raw stuffed chicken products rely on consumers’ ability to identify them as raw, to read and recall cooking instructions, to adequately cook the products according to validated cooking instructions, typically in conventional ovens, and to verify the product’s internal temperature using a food thermometer. Results from this survey highlight possible challenges consumers face preparing these products safely and the need for additional action. Given the substantial percentage of respondents who reported using an appliance other than an oven, and socioeconomic characteristics of respondents with lower oven usage, companies could consider implementing additional interventions that rely less on labeling and consumer preparation practices and instead control or reduce levels of *Salmonella* in these products, such as selling them fully cooked, or monitoring and testing *Salmonella* levels, to ensure safety.

The findings in this report are subject to at least four limitations. First, responses were self-reported and therefore subject to recall and social desirability biases. Second, although weighted to represent the U.S. population, the survey sample might not be representative. Third, the survey did not specify raw frozen stuffed chicken products; therefore, consumers possibly reported appliances that they use to prepare fully cooked stuffed chicken products. However, previous studies indicate that some consumers might be unaware that these products are usually raw (*2*). Finally, the survey did not include questions about whether cooking instructions were noticed or followed, or which appliances respondents owned; therefore, reasons that specific appliances were used could not be assessed.

Although *Salmonella* has not historically been considered an adulterant in not-ready-to-eat products, including raw frozen stuffed chicken products, the U.S. Department of Agriculture Food Safety and Inspection Service recently announced its intention to declare it an adulterant in these products (*10*). These findings can guide regulatory policy and prevention strategies for the industry.

**Summary**

**What is already known about this topic?**

Frozen stuffed chicken products remain a source of *Salmonella* outbreaks despite changes to packaging instructing consumers to cook these products in ovens and to avoid using microwaves.

**What is added by this report?**

More than one half of respondents to an Internet panel survey reported using an appliance other than an oven to cook frozen stuffed chicken products; 29% used a microwave. Respondents with lower incomes and who live in mobile types of homes reported lower oven use and higher microwave use.

**What are the implications for public health practice?**

Economic and other factors might influence access to recommended cooking appliances. Companies could consider implementing interventions that rely less on labeling and consumer preparation practices to ensure safety.

## Footnotes

* These authors contributed equally to this report.

†
https://www2.census.gov/geo/pdfs/maps-data/maps/reference/us_regdiv.pdf

§ 45 C.F.R. part 46.102(l)(2), 21 C.F.R. part 56; 42 U.S.C. Sect. 241(d); 5 U.S.C. Sect. 552a; 44 U.S.C. Sect. 3501 et seq.

¶ Housing that is occupied without payment of rent could include housing owned by friends or relatives who live elsewhere and who allow occupancy without charge or could include housing provided as compensation for persons such as caretakers, ministers, tenant farmers, or others.
